# Predicting Parallelism and Quantifying Divergence in Microbial Evolution Experiments

**DOI:** 10.1128/msphere.00672-21

**Published:** 2022-02-09

**Authors:** William R. Shoemaker, Jay T. Lennon

**Affiliations:** a Department of Biology, Indiana University, Bloomington, Indiana, USA; University of Michigan—Ann Arbor

**Keywords:** experimental evolution, microbial evolution, evolution, parallel evolution, adaptation

## Abstract

The degree to which independent populations subjected to identical environmental conditions evolve in similar ways is a fundamental question in evolution. To address this question, microbial populations are often experimentally passaged in a given environment and sequenced to examine the tendency for similar mutations to repeatedly arise. However, there remains the need to develop an appropriate statistical framework to identify genes that acquired more mutations in one environment than in another (i.e., divergent evolution), genes that serve as genetic candidates of adaptation. Here, we develop a mathematical model to evaluate evolutionary outcomes among replicate populations in the same environment (i.e., parallel evolution), which can then be used to identify genes that contribute to divergent evolution. Applying this approach to data sets from evolve-and-resequence experiments, we found that the distribution of mutation counts among genes can be predicted as an ensemble of independent Poisson random variables with zero free parameters. Building on this result, we propose that the degree of divergent evolution at a given gene between populations from two different environments can be modeled as the difference between two Poisson random variables, known as the Skellam distribution. We then propose and apply a statistical test to identify specific genes that contribute to divergent evolution. By focusing on predicting patterns among replicate populations in a given environment, we are able to identify an appropriate test for divergence between environments that is grounded in first principles.

**IMPORTANCE** There is currently no universally accepted framework for identifying genes that contribute to molecular divergence between microbial populations in different environments. To address this absence, we developed a null model to describe the distribution of mutation counts among genes. We find that divergent evolution within a given gene can be modeled as the absolute difference in the total number of mutations observed between two environments. This quantity is effectively captured by a probability distribution known as the Skellam distribution, providing an appropriate statistical test for researchers seeking to identify the set of genes that contribute to divergent evolution in microbial evolution experiments.

## OBSERVATION

Biologists have long been fascinated by the degree to which evolution is repeatable (1). Independently evolving microbial populations frequently evolve similar genotypes and phenotypes, a phenomenon known as parallel evolution (2, 3). Through the rise of evolve-and-resequence experiments as high-throughput screens for adaptation, researchers can now identify recurrent mutations across replicate populations to pare down the vast number of potentially adaptive mutations into those that putatively confer the largest fitness benefits (4, 5). Furthermore, evolve-and-resequence experiments have revealed that the outcomes of evolution are often conditional on ancestral genotype (6–10) as well as the environment in which the microbial populations were maintained (11–16), a phenomenon known as divergent evolution.

Despite the potential power of evolve-and-resequence experiments, statistical frameworks to quantify the repeatability of evolution are lacking. In recent years, models that coarse-grain over molecular details have been remarkably successful in identifying general evolutionary principles (17). This approach, and the underlying motivations to develop straightforward interpretations of biological phenomena, raises the question of whether there are intuitive ways in which the contributors to divergent evolution can be identified. To address this task, we first determined the extent to which patterns of parallel evolution at the gene level can be predicted using a statistical model containing zero free parameters with publicly available data. Building on these results, we formulated and tested an interpretable null model of divergent evolution at the gene level. In both cases, we use data from published experiments with bacteria, but in principle, the statistical methods can be applied to populations of archaea, microeukaryotes, and viruses.

### Predicting genetic parallelism among replicate populations.

**FIG 1:**
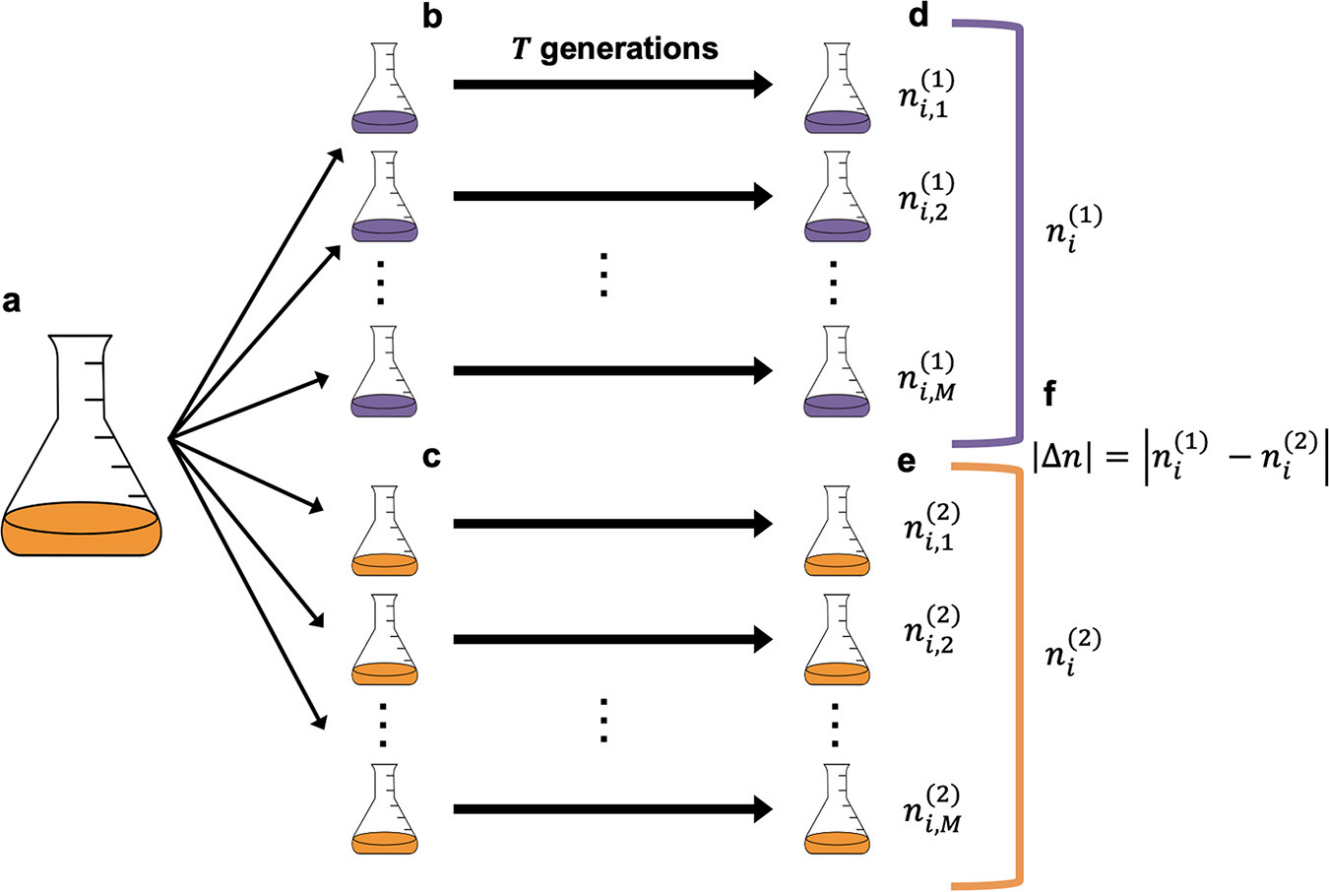
(a) A typical evolve-and-resequence experiment is performed by splitting a culture that has been grown from a single colony, inoculating cells into replicate flasks constituting one or more environmental conditions (e.g., purple or orange), and propagating the population over time by periodically transferring cells into new flasks with fresh medium. (b and c) After a given number of generations has elapsed, replicate populations are often sequenced, allowing the number of *de novo* mutations at a given gene to be calculated. (d to f) The degree of parallel evolution within each environment is quantified by taking the sum of mutation counts across replicate populations for a given gene (d and e), while the degree of divergent evolution is quantified by taking the absolute difference in mutation counts between environments (|Δ*n*|) (f).

The task of identifying genes that contribute to divergent evolution can be viewed as the equivalent of identifying genes that undergo a greater degree of parallel evolution in one environment than in another environment (Fig. 1). This observation suggests that it is necessary to first identify an appropriate model of parallel evolution within a single environment in order to develop a null model of divergence. Given that the per-generation probability of acquiring a mutation at a given gene is low and the number of generations is large, it is reasonable to assume that a given gene acquires mutations as a Poisson process. We can model the sampling distribution of this process as the probability of observing *n_i_*_,_*_j_* mutations in the *i*th gene within a population that acquired a total of *n*_tot,_*_j_* mutations:

(1) $$P\text{(}n_{i,j}|n_{\text{tot},j})\;=\;\left(\begin{array}{c} n_{\text{tot},j}\\ n_{i,j} \end{array}\right){\left(\frac{n_{i,j}}{\sum_{i}n_{i,j}}\right)}^{n_{i,j}}{\left(\frac{\sum_{k\neq i}n_{k,j}}{\sum_{i}n_{i,j}}\right)}^{n_{\text{tot},j}-n_{i,j}}$$

We can then determine whether we can predict statistical patterns from empirical data using equation 1. Given that mutation count data from evolve-and-resequence experiments are often sparse (i.e., zeros comprise a large proportion of the observations), it is natural to calculate the proportion of populations that have at least one mutation in a given gene (i.e., occupancy, *o_i_* [18]) and compare our empirical estimate to an expected value by averaging over *M* replicate populations:

(2) $$o_{i}=1-\frac{1}{M}\overset{M}{\underset{j}{\sum }}P\left(0|n_{\text{tot},j}\right)=1-\frac{1}{M}\overset{M}{\underset{j}{\sum }}{\left(\frac{\sum_{k\neq i}n_{k,j}}{\sum_{i}n_{i,j}}\right)}^{n_{\text{tot},j}}$$

**FIG 2:**
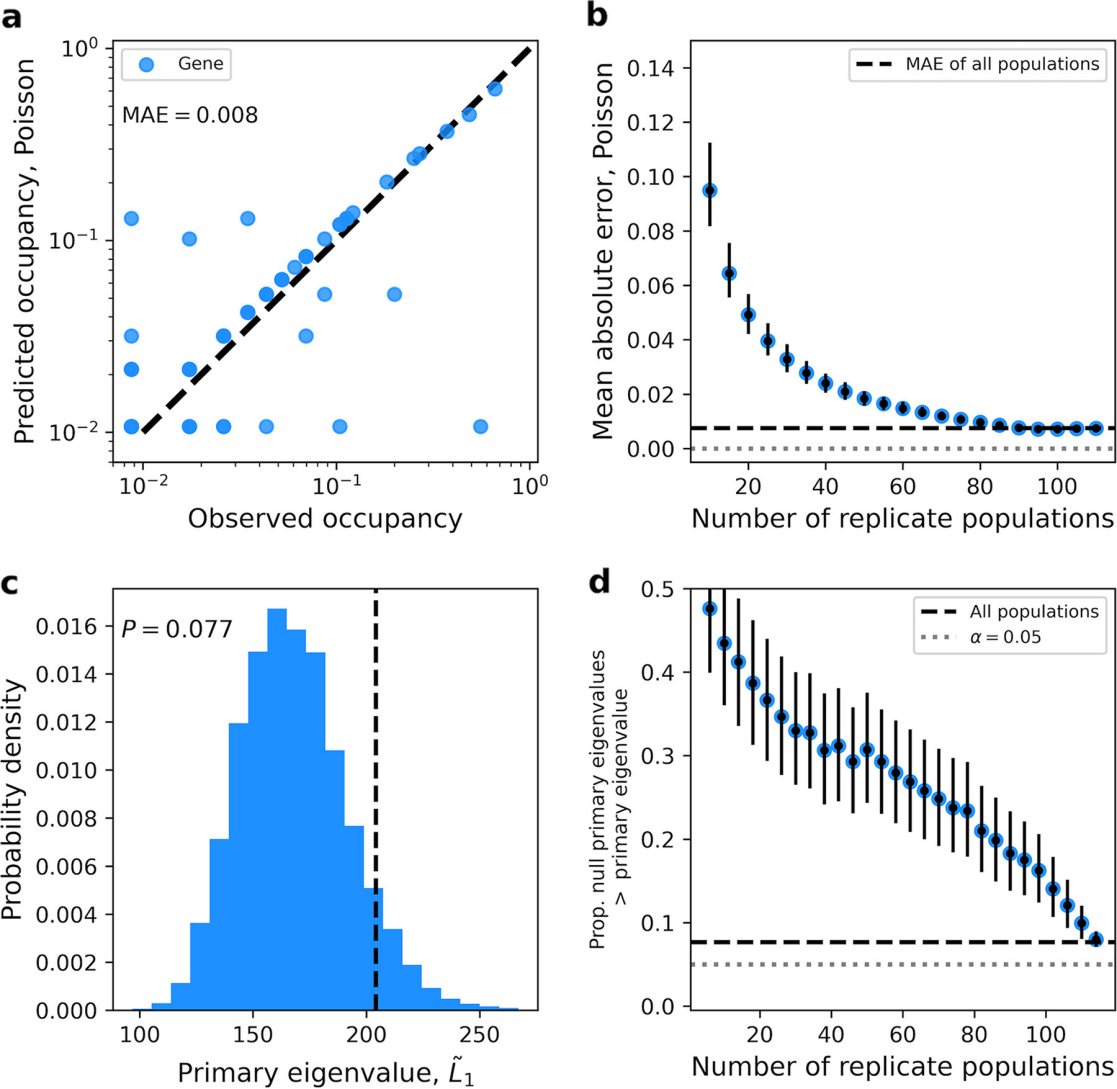
(a) Using the Poisson distribution, we were able to predict the occupancy of nonsynonymous mutations for a given gene among 115 replicate E. coli populations. (b) Using the same data set, we were able to subsample replicate populations to examine how the level of error in our prediction decreased as the number of replicate populations increased. (c) The degree of covariance between genes is summarized by the primary eigenvalue of the gene-by-population matrix of mutation counts (dashed black line). By generating null count matrices, we simulated a null distribution of primary eigenvalues to calculate the *P* value for the observed degree of covariance. (d) Similar to the analysis in panel c, we examined how the ability to detect covariance changes as the number of replicate populations increases by calculating the fraction of observed primary eigenvalues greater than the null.

To test this prediction, we calculated 〈*o_i_*〉 from empirical data. Given that the number of genes (i.e., variables) is typically much larger than the number of mutations (i.e., observations) within a typical population in an evolve-and-resequence experiment, it is necessary to examine an experiment that maintained a large number of replicates. The Escherichia coli evolve-and-resequence data set from Tenaillon et al. contains 115 replicate populations that originated from a single genotype (i.e., CFU) and were passaged for 2,000 generations (11), a number that was, and still is, far larger than that of a typical evolve-and-resequence experiment. We found that our model does a reasonable job capturing the observed occupancy of nonsynonymous mutations (Fig. 2a), with a mean absolute error (MAE) of ∼0.008. The success of the Poisson model is even more apparent when it is compared to a reasonable alternative model where each lineage can acquire a maximum of one mutation in a given gene (see Text S1, equation S2, and Fig. S1 in the supplemental material). However, while the MAE decreased with an increasing number of replicate populations, it ultimately saturated (Fig. 2b). The fact that it does not reach zero suggests that features not incorporated into our model, such as nonindependence among genes, may be necessary to fully explain the distribution of mutation counts.

10.1128/msphere.00672-21.1 TEXT S1 Derivation of occupancy for a geometric distribution. Download Text S1, DOCX file, 0.01 MB. Copyright © 2022 Shoemaker and Lennon. 2022 Shoemaker and Lennon. https://creativecommons.org/licenses/by/4.0/ This content is distributed under the terms of the Creative Commons Attribution 4.0 International license.

10.1128/msphere.00672-21.2 FIG S1 As a contrast to our model where the number of mutations at given gene is the result of an independent Poisson process, we examined whether a geometric distribution provided a reasonable alternative. (a) By estimating the observed occupancy and comparing it to the expected occupancy described in equation S2, we found that the geometric distribution provided a poor quantitative explanation for the distribution of occupancies of nonsynonymous mutations for a given gene among 115 replicate E. coli populations. (b) By comparing the relative error of the geometric distribution to that of the Poisson process, it is clear that the Poisson model is the superior model, as its prediction has a lower error rate for the vast majority (∼97%) of genes. Download FIG S1, TIF file, 2.8 MB. Copyright © 2022 Shoemaker and Lennon. 2022 Shoemaker and Lennon. https://creativecommons.org/licenses/by/4.0/ This content is distributed under the terms of the Creative Commons Attribution 4.0 International license.

To determine whether nonindependence among genes was necessary to incorporate in our model, we tested whether we could detect signals of covariance in our data. Because the number of genes that acquired mutations in an experiment can be in the hundreds, and mutation count data are sparse, attempting to estimate individual covariances for all pairs of genes would be unreasonable. Instead, we estimated a global signature of covariance and compared it to an appropriate null distribution (see “Predicting and quantifying parallelism,” below). While the global signal of covariance increased with the number of replicate populations, it was weak for values typical of most evolution experiments (5 to 20 populations) (Fig. 2c and d) and was only borderline significant when all 115 replicate populations were included (*P* = 0.072). This result suggests that we can proceed with the development of a null model of divergent evolution without the incorporation of covariance between genes, instead modeling the degree of parallel evolution for a given gene as an independent random variable.

### Identifying genes that contribute to divergent evolution between environments.

The success of the multivariate Poisson model in describing the distribution of mutation counts within a given environment along with the overall weak signals of covariance provide justification for modeling the distribution of mutation counts among genes as an ensemble of effectively independent variables. We can then model divergent evolution at a given gene as the difference between two independent Poisson rates. In terms of mutation counts, we can identify the meaningful variable as the absolute difference in mutation counts between two environments for a given gene ($\left|\Delta n_{i}\right|=\;\left|n_{i}^{\left(1\right)}-n_{i}^{\left(2\right)}\right|$). The distribution of |Δ*n*| has been previously derived and is known as the Skellam distribution (19). Starting with the null Poisson rates for each treatment ($\lambda_{1}=n_{\text{tot}}^{\left(1\right)}/N_{\text{genes}}$; $\lambda_{2}=n_{\text{tot}}^{\left(2\right)}/N_{\text{genes}}$), we define the probability mass function of the absolute value of |Δ*n*| as

(3) $$\mathrm{Pr}\text{[}\left|\Delta n\right|{\;|\lambda }_{1},{\;\lambda }_{2}]=\{\begin{array}{c} e^{-(\lambda_{1}+\lambda_{2})}\left[{\left(\frac{\lambda_{1}}{\lambda_{2}}\right)}^{\frac{\left|\Delta n\right|}{2}}I_{\Delta n}\left(2\sqrt{\lambda_{1}\lambda_{2}}\right)+{\left(\frac{\lambda_{2}}{{\text{λ}}_{1}}\right)}^{\frac{\left|\Delta n\right|}{2}}I_{-\Delta n}\left(2\sqrt{\lambda_{1}\lambda_{2}}\right)\right]\text{if}\;\left|\Delta n\right|>\;0\;\\ \;e^{-\left(\lambda_{1}+\lambda_{2}\right)}I_{0}\left(2\sqrt{{\text{λ}}_{1}\lambda_{2}}\right)\;\text{if}\;\left|\Delta n\right|=0 \end{array}\;$$

Where *I*_Δ_*_n_*(·) is a modified Bessel function of the first kind. Building on a previous approach developed to identify contributors of parallel evolution (20), we can define the *P* value as

(4) $$P_{i}=\underset{\left|\Delta n\right|\;\geq \;\left|\Delta n_{i}\right|}{\sum }\mathrm{Pr}\text{[}\left|\Delta n\right|{\;|\lambda }_{1},{\;\lambda }_{2}]$$

To reduce the number of tests, we can calculate *P* values only for |Δ*n*| ≥ *n*_min_, where the expected number of genes with |Δ*n*| ≥ *n*_min_ and *P_i_* ≤ *P* is

(5) $$\bar{N}\left(P\right)\approx \;\overset{N_{\text{genes}}}{\underset{i=1}{\sum }}\overset{\infty }{\underset{\left|\Delta n\right|=n_{\text{min}}}{\sum }}\theta (P-P_{i}(\left|\Delta n\right|))\cdot \mathrm{Pr}\text{[}\left|\Delta n\right|{\;|\lambda }_{1},{\;\lambda }_{2}]$$

where θ(·) is the Heaviside step function. We can then compare this number to the observed number of genes, *N*(*P*), defining a critical *P* value (P*) for a given false discovery rate (FDR), α, as

(6) $$\frac{\bar{N}\left(P^{\text{*}}\right)}{N\left(P^{*}\right)}\leq \alpha$$

To apply this approach, it was necessary to identify an evolve-and-resequence experiment that maintained at least two treatments. We identified an appropriate experiment where six replicate populations of the bacterium Burkholderia cenocepacia were propagated for ∼600 generations in four environments, allowing us to identify the set of genes that were consistently enriched for nonsynonymous mutations within a given treatment across all pairwise treatment comparisons (12) (Table S1). Our results largely agree with the conclusions of the original study: virtually all the genes that were significantly enriched within a single treatment in the original study were also identified as contributors to environment-specific adaptation (12).

10.1128/msphere.00672-21.3 TABLE S1 Using equation 4, we calculated a *P* value of divergent evolution for each pairwise treatment comparison for each gene. To identify candidates of adaptation that are unique to a given treatment, we identified the set of genes that were significantly enriched for nonsynonymous mutations within a given treatment for all pairwise treatment comparisons. Download Table S1, TIF file, 0.6 MB. Copyright © 2022 Shoemaker and Lennon. 2022 Shoemaker and Lennon. https://creativecommons.org/licenses/by/4.0/ This content is distributed under the terms of the Creative Commons Attribution 4.0 International license.

### Concluding remarks.

We investigated the distribution of mutation counts in bacterial evolve-and-resequence experiments. We found that a Poisson model containing zero free parameters sufficiently explained the distribution of mutation counts across genes. This result suggests that parallel evolution among replicate populations in evolve-and-resequence experiments can be quantitatively predicted without the use of models that require statistical fits (e.g., linear regression). We then developed an intuitive null model for identifying genes that contributed to the genetic differences that accrued between bacterial populations that evolved in different environments (i.e., divergent evolution). Using this result, the difference in the numbers of mutations within a given gene between treatments (|Δ*n*|) can be modeled as a difference in Poisson rates between treatments (i.e., the Skellam distribution).

Our approach should be robust to documenting parallel and divergent evolution in evolve-and-resequence experiments with diverse microbial taxa. While we focused on bacterial case studies owing to features related to experimental design, there is no reason why the framework cannot be applied to archaea, microeukaryotes, and viruses. One biological feature that may require additional consideration is the existence of sexual recombination in eukaryotes. However, this is unlikely to substantially alter our results, as recombination breaks the physical linkage between mutations, which subsequently reduces the magnitude of covariance between a given pair of genes. In addition, we note that the facilitation of recombination is the principal effect of sexual reproduction on the molecular evolutionary dynamics of a population, an evolutionary force that often occurs in bacteria. While we did not determine the extent to which the molecular details of recombination affect the accuracy of the Poisson model, and recombination rates are difficult to infer in bacterial evolve-and-resequence experiments, we note that there was evidence of homologous recombination in the experimental data that we examined (11), suggesting that the presence or absence of recombination alone is insufficient to substantially affect our predictions.

### Data.

To determine the degree to which we can predict statistical patterns of parallel evolution, we used a publicly available data set of one of the largest microbial evolve-and-resequence experiments. In this experiment, 115 replicate populations of Escherichia coli were serially transferred for 2,000 generations at 42.2°C (11). A single colony was isolated from each replicate population and sequenced at the end of the experiment.

To apply our divergence test, we used a publicly available data set from a factorially designed experiment where the bacterium Burkholderia cenocepacia was propagated for ∼600 generations at 37°C in a roller drum. Specifically, replicate populations (*n* = 6) were grown in either low- or high-carbon medium in the presence or absence of a bead that was used to promote biofilm or planktonic growth, respectively.

### Predicting and quantifying parallelism.

To test for a global signal of covariance between genes, we merged all nonsynonymous mutations from all replicate populations into a population-by-gene-count matrix. To account for gene size as a covariate, we corrected the number of mutations by calculating the excess number of mutations (i.e., multiplicity), $m_{i,j}=n_{i,j}\cdot \bar{L}/L_{i}$, where *L_i_* is the number of nonsynonymous sites in the *i*th gene and $\bar{L}$ is the mean of all genes in the genome (20). To determine whether covariance could be reliably detected at a given level of replication, we estimated the largest normalized eigenvalue over the set of eigenvalues ({*E*_1_}) (21, 22), defined as

(7) $${\tilde{e}}_{1}=\;\frac{e_{1}-\mu (M,\;N_{\text{genes}})}{\sigma (M,\;N_{\text{genes}})}$$

where *e*_1_ is normalized as $e_{1}=ME_{1}/\overset{M}{\underset{m=1}{\sum }}E_{m}$ to sum to *M*, *E*_1_ is the largest eigenvalue, and

(8) $$\text{μ}\left(M,\;N_{\text{genes}}\right)=\frac{{\left(\sqrt{N_{\text{genes}}-1}+\sqrt{M}\right)}^{2}}{N_{\text{genes}}}$$

(9) $$\sigma \left(M,\;N_{\text{genes}}\right)=\frac{\sqrt{N_{\text{genes}}-1}+\sqrt{M}}{N_{\text{genes}}}{\left(\frac{1}{\sqrt{N_{\text{genes}}-1}}+\frac{1}{\sqrt{M}}\right)}^{\frac{1}{3}}$$

As *M*, *N*_genes_ → ∞ and *N*_genes_/*M* → γ ≥ 1, *ẽ*_1_ tends toward the Tracy-Widom distribution (22, 23), although these limits can be relaxed (21, 24). A null distribution of *ẽ*_1_ was obtained by randomizing combinations of mutation counts constrained on the total number of mutations acquired within each gene across treatments and the number of mutations acquired within each treatment. Randomization was performed using a Python implementation of the ASA159 algorithm (25, 26).

### Data availability.

Instructions and code to reproduce our analyses are on GitHub (https://github.com/LennonLab/ParEvol). All processed data are available on Zenodo (https://zenodo.org/record/3779341).
