# Supplementary material for: Predicting Parallelism and Quantifying Divergence in Microbial Evolution Experiments
Source: mSphere. 2022 Feb 9;7(1):e00672-21. doi: 10.1128/msphere.00672-21 (PMC8826959; doi:10.1128/msphere.00672-21)
Supplement: TEXT S1 [file msphere.00672-21-s0001.docx]

**SUPPLEMENTAL TEXT S1**

***Saturation of fitness benefits***

As an alternative model to the Poisson distribution, we considered the case where a single nonsynonymous mutation saturates the fitness benefit provided by a given gene. This process can be readily modeled as a geometric distribution, where a given gene is effectively "waiting" to acquire a mutation, where the probability of acquiring a mutation at a given gene is equal to the fraction of mutations it acquired.

S1) $P_{\mathrm{geom}}\left( n_{i,j}>0 | n_{\mathrm{tot},j} \right)= \left( 1-\frac{\sum_{k\neq i} n_{k,j}}{\sum_{i} n_{i,j}} \right)^{n_{\mathrm{tot},j}}\cdot\frac{\sum_{k\neq i} n_{k,j}}{\sum_{i} n_{i,j}}$

from which we can define the expected occupancy as

S2) $\left\langle o_{i} \right\rangle^{\mathrm{geom}}=\frac{1}{M}\sum_{j}^{M} P_{\mathrm{geom}}\left( n_{i,j}>0 | n_{\mathrm{tot},j} \right)=\frac{1}{M}\sum_{j}^{M} \left( 1-\frac{\sum_{k\neq i} n_{k,j}}{\sum_{i} n_{i,j}} \right)^{n_{\mathrm{tot},j}}\cdot\frac{\sum_{k\neq i} n_{k,j}}{\sum_{i} n_{i,j}}$

We find that Eq. S2 does a comparatively poor job predicting the occupancy of genes (Fig. S1), with a substantially higher mean absolute error of $\approx0.023$. Furthermore, the genes where our predictions were worse using Eq. S2 were also worse using Eq. 2, meaning that the saturating effect model did not outperform the Poisson distribution for any gene.
