# Supplementary figures and images for: Predicting Parallelism and Quantifying Divergence in Microbial Evolution Experiments
Source: mSphere. 2022 Feb 9;7(1):e00672-21. doi: 10.1128/msphere.00672-21 (PMC8826959; doi:10.1128/msphere.00672-21)

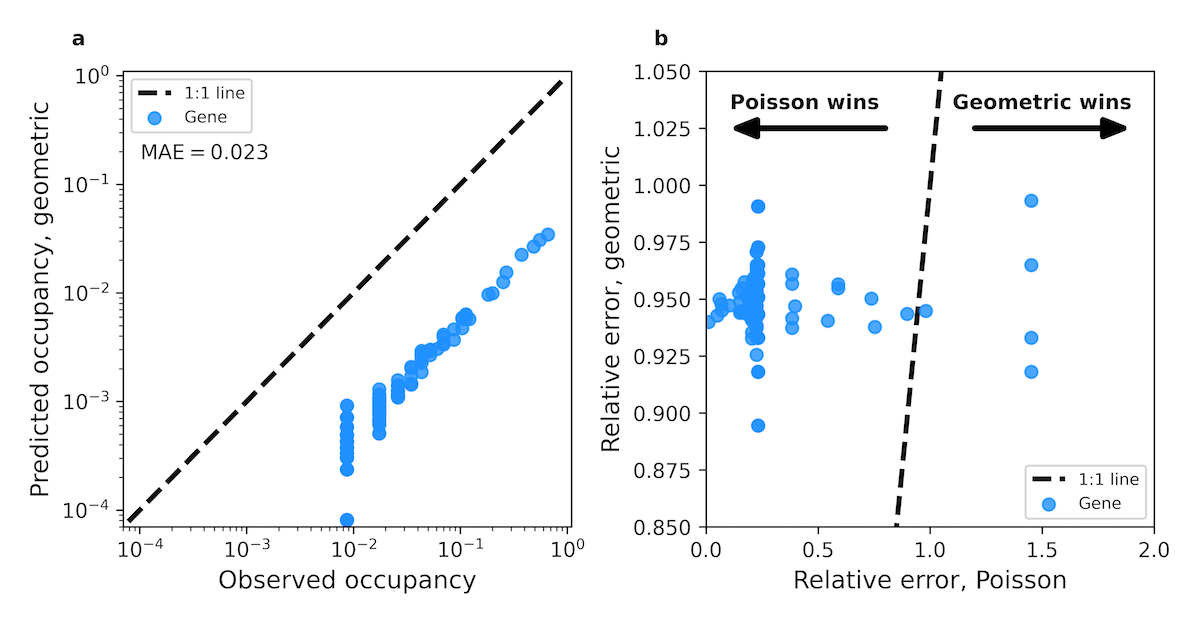

Supplement: FIG S1 [file msphere.00672-21-sf001.tif]

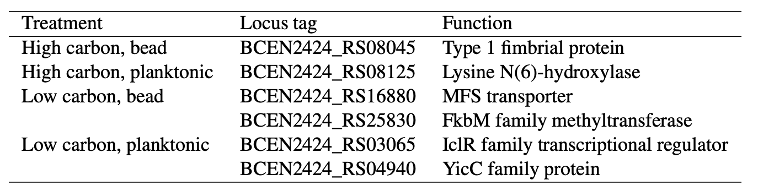

Supplement: TABLE S1 [file msphere.00672-21-st001.tif]
